# Structural modifications due to interface chemistry at metal-nitride interfaces

**DOI:** 10.1038/srep17380

**Published:** 2015-11-27

**Authors:** S. K. Yadav, S. Shao, J. Wang, X.-Y. Liu

**Affiliations:** 1 Materials Science and Technology Division, MST-8, Los Alamos National Laboratory, Los Alamos, New Mexico 87545, USA; 2 Department of Mechanical and Materials Engineering, University of Nebraska-Lincoln, Lincoln, NE 68583, USA

## Abstract

Based on accurate first principles density functional theory (DFT) calculations, an unusual phenomenon of interfacial structural modifications, due to the interface chemistry influence is identified at two metal-nitride interfaces with strong metal-nitrogen affinity, Al/TiN {111} and Al/VN {111} interfaces. It is shown that at such interfaces, a faulted stacking structure is energetically preferred on the Al side of the interface. And both intrinsic and extrinsic stacking fault energies in the vicinity Al layers are negligibly small. However, such phenomenon does not occur in Pt/TiN and Pt/VN interfaces because of the weak Pt-N affinity. Corresponding to structural energies of metal-nitride interfaces, the linear elasticity analysis predicts characteristics of interfacial misfit dislocations at metal-nitride interfaces.

Metal/ceramic interfaces are of great importance both scientifically and technologically^1^,^2^,^3^. Recently, multilayered metal/nitride at nanoscales received much attention due to their unique mechanical properties during deformations^4^,^5^,^6^,^7^,^8^. At 5 nm or below, enhanced plastic co-deformation in Al/TiN is observed^6^,^7^. Such co-deformation provides possibility of enabling design of novel metal/ceramic composites with high hardness and measureable ductility. Plastic deformations in both metal and ceramic layers are carried over by lattice dislocations that nucleated from interfaces. During deformation processes, these lattice dislocations propagate inside layers and are deposited on the interfaces. These deposited dislocations interact with interfaces and interface misfit dislocations. Thus it is essential to understand interface structure, interface properties and characters of interface misfit dislocations^9^,^10^,^11^,^12^.

Structural transformations at interfaces in crystalline materials are of significant fundamental interest as these transformations often lead to important implications in the material properties^13^. Interfacial structural changes or phase transformations have been observed as “complexions” in pure ceramics^14^ and metallic grain-boundaries^13^. However, research on the interfacial structural changes on the metal/ceramic interfaces is very limited^11^,^15^,^16^. Atomistic simulations with empirical interatomic potentials have been demonstrated to be reliable in exploring structure and properties of an interface. However, there is lack of accurate empirical potentials for metal-nitride interface. In literature, many studies using density functional theory (DFT) have been carried to understand the atomic structure, work of adhesion, electronic property and stability of range of metal/nitride interfaces^17^,^18^,^19^,^20^,^21^,^22^,^23^,^24^,^25^,^26^.

In this report, we investigated metal-nitride interfaces in metal-ceramics multilayers using accurate first principles DFT calculations. For the first time, an unusual phenomenon of interfacial structural modifications, due to the interface chemistry influences, is identified at metal/nitride, *i.e.*, Al/TiN and Al/VN {111} interfaces while no such structural modifications at Pt/TiN and Pt/VN interfaces. This drastic difference is ascribed to the metal-N affinity at the interface. The presence of nitrogen (N) atoms at interfaces changes the generalized stacking fault (GSF) energy landscape of Al layers nearby the interface in a significant way associated with the strong Al-N affinity at the interface, leading to such structural modifications. Finally, we predict characters of misfit interfacial dislocations at metal-nitride interfaces based on linear elasticity analysis.

## Results

Experimentally, it has been shown that, for Al/TiN multilayers, the orientation relation between the Al and TiN layers is {111}Al||{111}TiN at the interface, and the growth direction is along <111>^7^. TiN is in rock-salt (B1) crystal structure with mixed covalent, ionic and metallic bondings, with both Ti and N in face-centered cubic (fcc) sublattices. The {111} planes of TiN consist of alternating layers of Ti and N atoms. It has been shown before, at the Al/TiN interface, the preferred TiN (111) termination depends on the nitrogen chemical potential and that under usual experimental growth conditions, the N termination is the preferred interface^27^. Corresponding to a lattice mismatch of 4.95% (or 4.42% in DFT)^28^,^29^, semi-coherent interface forms and consists of coherent interface structures and misfit dislocation networks. According to the crystallographic analysis of the interface, there are several coherent interface structures that may have different excess potential energies and thermal stability^30^,^31^, such as normal fcc, intrinsic stacking fault, extrinsic stacking fault, and even high energy stacking fault structures. Correspondingly, characters of misfit dislocations are strongly related to these coherent interface structures^32^.

The thermally stable coherent interface structures can be identified based on the concept of GSF energy and the so-called γ surface. GSF energy, which was first introduced by Vitek^33^ about 40 years ago, is defined as the extra energy per unit area needed for a rigid shear displacement at a given glide plane. When the displacements are two-dimensional in a certain plane, the energy landscape in the plane is called the γ surface. The wells in the γ surface are corresponding to thermally stable structures. In addition, GSF energy is also useful in identifying likely and unlikely dislocation dissociation reactions by distinguishing stable and unstable stacking faults^34^,^35^.

**Figure 1:**
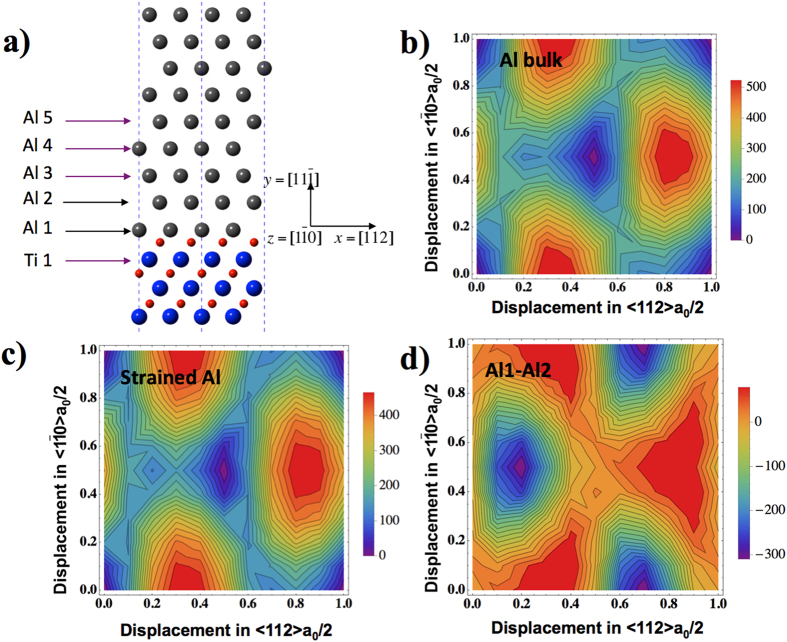
(**a**) The schematic of conventional bilayer atomic model of (111) Al/(111) TiN with N termination at the interface. (**b**) The γ surface computed for strain-free bulk Al, for (11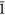) plane with displacements along [112] and [1
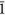
0], respectively. (**c**) The γ surface computed for Al strained: the Al lattice parameter is stretched to match that of TiN at the interface, for (11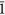) plane with displacements along [112] and [1
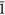
0], respectively. (**d**) The γ surface computed for Al1-Al2 planes at the Al/TiN interface in (**a**), with displacements along [112] and [1
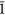
0], respectively. Units are in mJ/m^2^ in (**b**–**d**).

Figure 1(a) schematically shows an atomic model of Al/TiN (11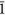) interface with fcc stacking sequence of *A*(Ti)*B*(Ti)*C*(Ti)*A*(Al) *B*(Al)*C*(Al). Figure 1(b–d) show the DFT computed γ surfaces of bulk Al, strained Al, and Al1-Al2 planes at Al/TiN (11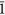) interface (see Fig. 1(a)). The displacements are along [112] and [1
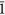
0] in the (11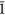) plane. For the strained Al, the lattice parameter of Al is biaxially strained to match that of TiN at the Al/TiN interface. It is clear from Fig. 1(b–d), the energy landscape of γ surface for the interfacial Al layers (Fig. 1(d)) is totally different than that of bulk Al or strained Al. The γ surface for bulk Al has a hexagonal symmetry since the {111} lattice plane of fcc Al, with the intrinsic stacking faults at the Shockley partial displacements, a_0_/6[112], a_0_/6[2
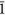
1], or a_0_/6[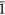
21]. Our DFT computed stacking fault energy is 140 mJ/m^2^, which is in excellent agreement with other calculations and experiments (earlier DFT value 142 mJ/m^2^ 36, and experiment value 162 mJ/m^2^ 37). For the biaxially strained Al, the γ surface changes only slightly by a reduction in the highest magnitude, while the shape of the γ surface maintains the same as that of bulk Al. For the interfacial Al layers, the γ surface also has the hexagonal symmetry, however, the minimum energy location on the γ surface is not at zero shift positions, but at the displacements of Shockley partial, a_0_/6<112> which are about 300 mJ/m^2^ lower in energy per area than the zero shift positions on the γ surface.

**Figure 2:**
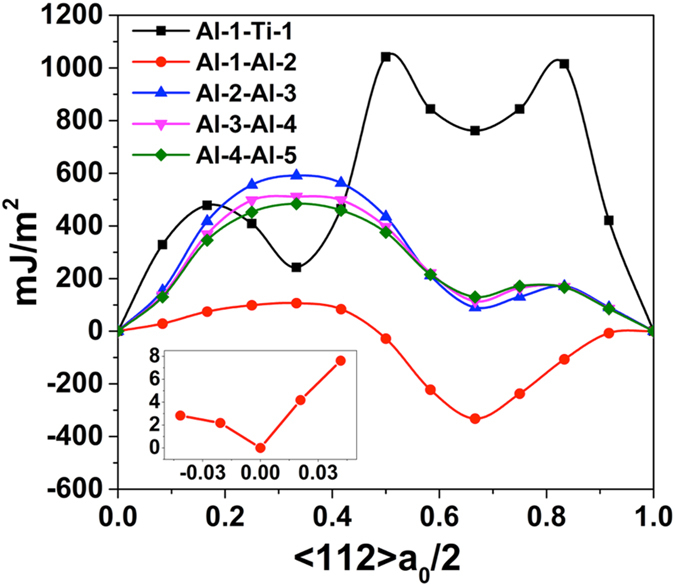
GSF energy curves for displacements in the (11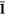) plane along <112> direction at the Al/TiN interface inFig. 1a. Inset shows the local minima in the GSF energy curve for Al1-Al2 planes.

Figure 2 shows various DFT computed GSF energy curves for different Al layers away from the interface plane, with displacements along [112] direction at the Al/TiN (11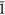) interface. The GSF energy curve for the Al-Ti plane shares a feature similar to that in TiN bulk, with the stable stacking fault in the “anti-twinning” sense^34^. In the GSF energy curves for Al layers, for Al1-Al2 planes, besides the lowest energy point at the a_0_/6[112] position, there is also another local energy minima at the zero shift position, only a few mJ/m^2^ in energy per area, as confirmed by our DFT calculations with different k-points samplings. This is shown as an inset in Fig. 2. Starting from Al2-Al3 planes, the GSF energy curves for Al layers farther away from the interface approach to that of bulk Al.

**Figure 3:**
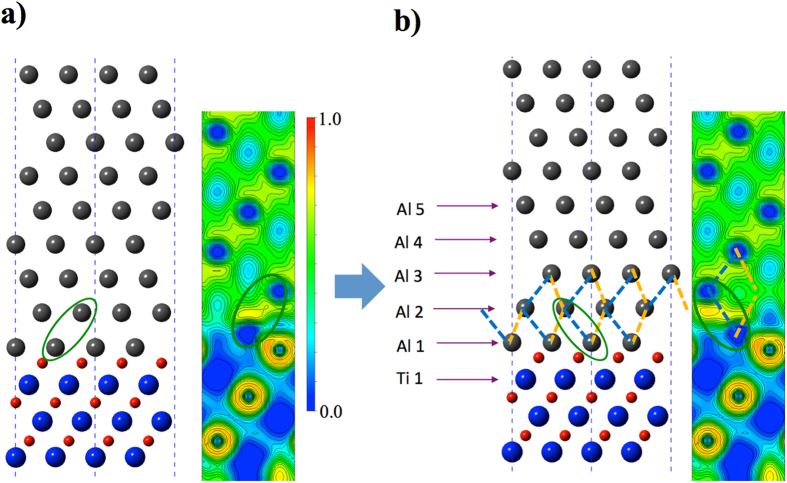
The structural transition from (a) no fault compared to (b) faulted structure between layers Al1 and Al2 at the Al/TiN interface. The electron localization functions are also shown.

The GSF calculations show that there is a structural modification to the faulted Al1-Al2 planes at the Al/TiN interface, as shown schematically in Fig. 3(a,b). Figure 3(a,b) also show the corresponding electron localization functions (ELF) from DFT simulations. Examination of the ELF density contours suggests that there is an electronic origin for such structural change at the interface. For the faulted interface structure, an extended electron density exists between Al1 and Al2 Al atoms, which reflects a stronger bonding than in the zero shift interface structure case. Such bonding change is possibly due to the influence from the underlying N dangling bonds at the interface.

**Figure 4:**
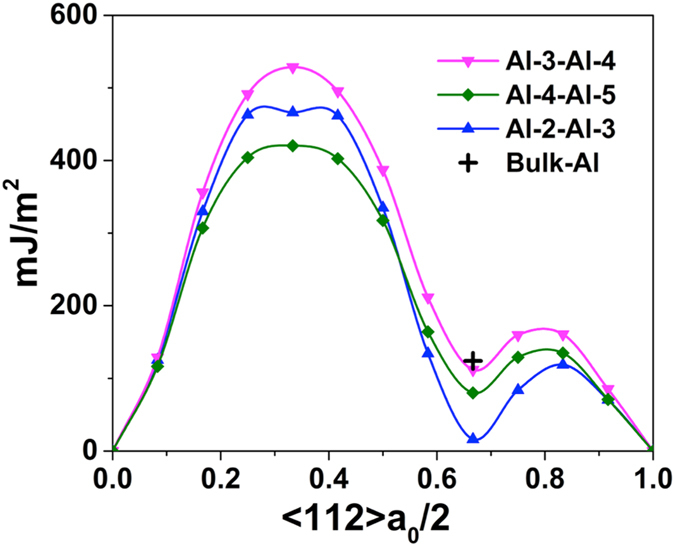
GSF energy curves for displacement in the (11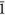) plane along <112> direction, at the faulted interface structure of the Al/TiN interface inFig. 3b. The stacking fault energy of strained bulk Al is also shown.

With the faulted interface structure as the starting structure, to understand the extent of the influence of N atoms at the interface, we recalculated the GSF energies of various Al planes away from the interfaces, again with displacements along [112] direction. Figure 4 shows the results. The stacking fault energy of strained Al is also shown for comparison. The most interesting part of Fig. 4 is that for the Al layers immediate above the faulted plane at the Al/TiN interface (Al2-Al3 planes), the stacking fault energy is extremely low, 16 mJ/m^2^, or about one tenth of the value in the bulk Al. We also computed the extrinsic stacking fault energy for the same Al layers, with DFT value of 12 mJ/m^2^ which is essentially degenerate with the intrinsic stacking fault energy value considering the DFT error bar of a few mJ/m^2^. The extremely low values of stacking fault energies implicate a strong tendency of the dissociation of full lattice dislocations into Shockley partials at the Al2-Al3 planes. The extremely low values of extrinsic stacking fault energies also implicate a more variety of different combinations of partial dislocations possible. As Al layers are further away from the interface, the GSF energies become higher, approaching, in an alternative way to the strained Al value. The faulted interface structure can also be viewed as a “local” twin at the interface. Lowering of stacking fault energies at the interface can enable twin nucleation at interfaces as well.

**Figure 5:**
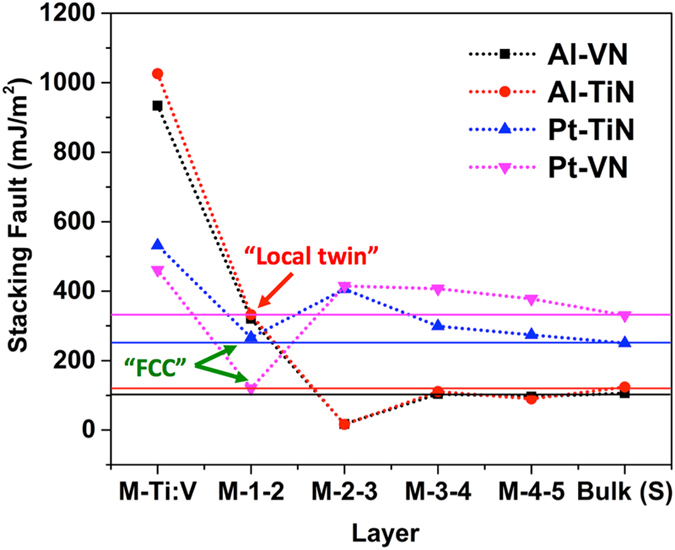
The stacking fault energies at different (11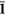) planes away from the (11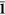) interface, along <112> direction, for different metal (Al, Pt) and nitrides (TiN, VN) interface systems. The stacking fault energies of strained bulk metals are also shown (solid lines).

To understand the chemical effect, a few different metal/nitride systems are explored. First, to understand if the transition metal in the nitride is contributing in a significant way, a different nitride, VN is used. VN also has rock-salt crystal structure^24^. To form a coherent interface of Al on the VN {111} plane, a lattice strain of 1.23% is used, as determined from DFT (experimental lattice misfit value is 2.23%)^24^. A comparison of the stacking fault energies at different planes away from the (11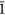) interface, for both Al/VN and Al/TiN interfaces, is shown in Fig. 5. The DFT computed Al/VN and Al/TiN results are very close, albeit the chemical difference of V and Ti. Such difference has appreciable influence only at the Al1-V(Ti) layers, which could be due to the different charge state of V and Ti in the nitride.

To make a change in the affinity between fcc metal atom and N, Pt is used to replace Al since it is known that Pt has low affinity with N^38^,^39^. Other metals like Cu also show low affinity with N^40^. However, the misfit strain between Cu and TiN is too large (>16%), so Cu is not used in this study. To form coherent interfaces between Pt and TiN or VN, lattice strains of 7.05% or 3.78% are used, from our DFT computations (experimental lattice misfit values are 8.16% and 5.36%)^41^. The lattice mismatch for the interface between Pt and TiN is somewhat large (7.05%), which may affects the electronic structure, a semicoherent interface structure may capture more accurately the interface properties. However, this is not carried out due to the limit of computational power. In Fig. 5, a comparison of the stacking fault energies at different planes away from the (11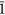) interface, for both Pt/VN and Pt/TiN interfaces, is shown. The stacking fault energies of strained Pt in the Pt/VN and Pt/TiN cases are also shown. For both interfaces, the metal layers away from the interface plane maintain fcc stacking as in the bulk metals. This is very different from the Al/VN and Al/TiN cases. The stacking fault energy for the Pt1-Pt2 layers has a modest decrease in value in the Pt/VN case while in the Pt/TiN case, very little change from the strained bulk value. It is also noted that for both Pt/VN and Pt/TiN cases, at the interface, Pt atoms in Pt1 layer are at “on top” sites of the underlying N layers (not shown). Such unique bonding is due to the weak bonding between Pt-N (low affinity) and it was also observed in the Cu/TiN interfaces in our earlier DFT study^27^.

## Discussion

The DFT simulations and electron density analysis confirmed that the bonding between the metal layers and the underlying nitrogen atoms plays the crucial role that resulted in the interfacial structural changes. Similar to that in TiN, the bonds between the metal layers and the underlying nitrogen atoms are expected to have a mixed covalent, ionic and metallic bonding nature, with the highly directional covalent bonding as the dominant one. It is then not so surprising that as transitional layers (Al1-Al2 layers) experiencing the transition from the ceramic bonds to the metallic bonds, electron density redistribution can cause structural ordering changes. The electronic origin determines that such effect is of short range in nature. And as a general rule, such effect may also exist in other types of layered materials, such as metal-oxide, or metal-carbide nanolayered composites.

**Figure 6:**
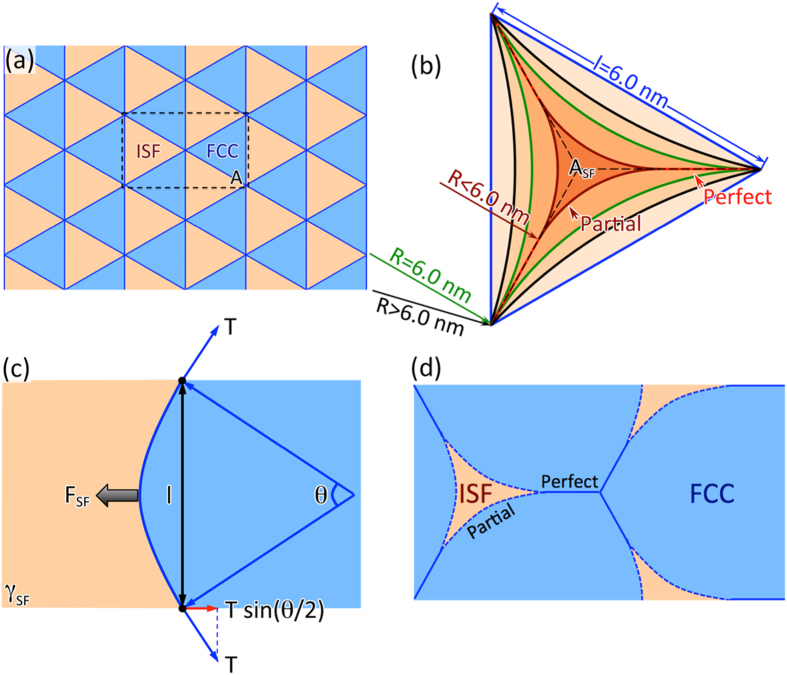
Schematic drawings that illustrate. (**a**) MDN structure with zero stacking fault energy; (**b**) the presence of curvature in partial dislocation lines and their possible recombination into perfect dislocations; **(c)** the balance between the stacking fault energy and the dislocation line tension; **(d)** an example structure of MDN within the periodic region marked in **(a)**, the stacking fault energy is relatively high so that the ISF regions have shrank and the partial dislocations have combined to form perfect dislocations.

The interface structure change, as demonstrated in the case of Al/TiN in this work, may influence the characteristics of interfacial misfit dislocations at metal-nitride interfaces. Using the linear elasticity theory, we predict characters of interfacial misfit dislocations at metal-nitride interfaces. Due to the large lattice mismatch between Al and TiN, the misfit dislocation network (MDN) (see Fig. 6(a)) exists between Al and TiN. The spacing of the dislocations is determined to be 5.2 nm, and the spacing between the intersections (nodes) 6.0 nm. The positions of MDN are determined by several factors. The MDN is usually a network of partials dislocation lines that separates the equilateral triangular regions of intrinsic stacking fault (ISF, brown in Fig. 6(a)) and perfect fcc stacking (FCC, cyan in Fig. 6(a))^30^,^31^. The system reduces its overall energy by curving the partial dislocation lines towards the stacking faults and therefore reducing the total stacking fault energy. Depending on the stacking fault energy, the dislocation lines have different curvatures. Figure 6(b) shows the variation of the area of a single stacking fault region with increasing stacking fault energy. If the stacking fault energy is zero, the dislocation lines have zero curvature (blue lines), and the stacking fault is a perfect equilateral triangle. When the stacking fault energy increases, the dislocation lines start to curve inwards (R > 6.0 nm, black lines) and reduce the area of the stacking fault. A critical point is reached when the curvature radius is equal to the edge length of the triangle (R = 6.0 nm, green lines), beyond which the partial dislocation lines start to recombine to form perfect dislocation lines (R < 6.0 nm, red and dark red lines correspond to perfect and partial dislocations). The curvature radius of the partial dislocation lines can be calculated by solving the force balance on the dislocation lines (Fig. 6(c)). The dislocation nodes serve as pinning points. Due to the positive stacking fault energy, the partial dislocation lines are pulled towards the stacking faults,


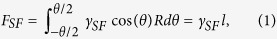


where *F*_SF_ is the resultant force on the dislocation line due to the stacking fault energy, *γ*_SF_ is the stacking fault energy (the unit is energy per unit area), *θ*, *R* and *l* are the central angle, the radius and the span of the arc, respectively. Note that when *R *≥ 6.0 nm, *l* = 6.0 nm. At the pinning points, the horizontal force component (2*T*sin(*θ*/2), where *T* = *αμb*^2^) equals to the force due to the stacking fault, such that


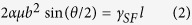


and


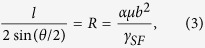


where *α* and *μ* are the constants used to estimate the dislocation line tension, which are explained in details in the methods section. At the critical point, *R* = *l* and *θ* = *60°*. The excess energy due to the presence of the misfit dislocations is calculated over a periodic region (dashed rectangle in Fig. 6(a)) of the MDN, an example of such a region with relatively high stacking fault energy is given in Fig. 6(d). In this case, the partial dislocation lines (dashed lines) have curvature smaller than 6.0 nm and perfect dislocation segments (solid lines) have formed. The excess energy of this region is therefore the sum of the total stacking fault energy in the ISF regions (brown), the line energy of the dislocations, and the coherent strain energy of Al monolayer between the MDN and the interface. We have calculated the excess energy of the MDN at different locations and the results are summarized in Table 1. From Table 1, it is found that the lowest excess energy of the MDN is when the MDN is located in Al2-Al3 plane (457 mJ/m^2^). The excess energies for MDN immediately above (Al3-Al4) or below (Al1-Al2) are 587 mJ/m^2^ and 583 mJ/m^2^, respectively. It is therefore expected that the partial dislocations network MDN be located in Al2-Al3 plane.

This in turn will also influence the dislocation interactions and slip resistance at interfaces, thus affecting the mechanical functionality of these novel composites. For example, similar to the case of twinning boundary, a lattice dislocation can approach the interface, and dissociate into partial dislocations, with one partial passing through the interface and the other partial sliding along the interface, thus affecting the ductility of the composites^42^,^43^.

In summary, based on accurate DFT calculations, an unusual phenomenon of interfacial structural modifications, due to the interface chemistry influences, is identified at metal/nitride, *i.e.*, Al/TiN and Al/VN {111} interfaces. And both intrinsic and extrinsic stacking fault energies in the vicinity Al layers are negligibly small. However, such phenomenon does not occur in Pt/TiN and Pt/VN interfaces because of the weak Pt-N affinity. This drastic difference is ascribed to the metal-N affinity at the interface. The presence of nitrogen atoms at interfaces changes the GSF energy landscape of Al layers nearby the interface in a significant way associated with the strong Al-N affinity at the interface, leading to such structural modifications. Finally, we predict characters of misfit interfacial dislocations at metal-nitride interfaces based on linear elasticity analysis. It is expected that the partial dislocations network MDN be located in Al layers where the intrinsic stacking fault energies are negligibly small.

## Methods

### Density Function Theory Calculations

The DFT calculations have been performed using the Vienna Ab initio Simulation Package (VASP)^44^, in which the Perdew, Burke, and Ernzerhof (PBE)^45^ generalized gradient approximation (GGA) exchange-correlation functional and the projector-augmented wave (PAW) method^46^ have been employed. For all the calculations, a plane wave cutoff of 500 eV for the plane wave expansion of the wave functions is used to obtain highly accurate forces. The electronic convergence tolerance is 10^−5 ^eV/atom. Force tolerance for the structural relaxation is 0.05 eV/Å. By reducing force tolerance for the structural relaxation from 0.05 eV/Å to 0.02 eV/Å, the stacking fault energies changes are less than 5 mJ/m^2^, smaller than the estimated DFT error of 10 mJ/m^2^. For all calculations 10 × 6 × 1 k-points are used. Accuracy of the results has already been established in our previous papers^27^,^47^,^48^. GSF energy is the excess energy per unit area, calculated by imposing a rigid shear displacement between two neighboring layers of atoms, except that all atoms are allowed to relax in the directions perpendicular to the shear displacement direction. In the case of shear between Al-Ti layers, the shear displacement can also be applied between two neighboring layers at the interface, while allowing the N layer in-between the two metal-atoms layers to relax. The calculation method is similar to our earlier work on TiN and MgO^34^. For the interface calculations, nine metal layers and six nitride layers are used in the supercell, which is periodic in the interface in x = [112] and z = [1
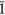
0] directions. In the direction perpendicular to the interface, y = [11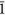] direction, surface slabs with a vacuum space of at least 1 nm are used to avoid the surface-surface interactions. The system is fixed in x and z directions during ionic relaxations while allowing relaxation in the surface slabs. Starting from the 5^th^ metal layer, the stacking fault energy approaches the bulk value. Adding another six layers of TiN in the supercell changes the stacking fault energies, typically by a few percent, in the range of 1–20 mJ/m^2^, which does not alter the trend in the observed changes and the resulting conclusions.

### Linear Elasticity Theory Calculations

The linear elasticity theory has been employed^31^ to calculate the excess energy (*γ*_exc._) due to the presence of misfit dislocation network and stacking faults as a function of their positions, e.g. precisely at the interface (between Al *1* and Ti *1*) vs. one or *n* (111) planes away from the interface within Al layers (between Al *n* and Al *n* + *1*). The excess energy is evaluated based on the contribution of three main components: the energy of the stacking faults, the line energies of the misfit dislocations, and the coherent strain energy in Al atomic monolayers between misfit dislocation network and the Al-TiN interface. Accordingly, the excess energy is calculated over a periodic region (dashed rectangle in Fig. 6(a)) of the MDN using 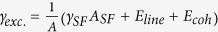. *A* is the area of the periodic region on the MDN. 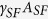 is the total excess energy of the stacking faults (brown regions); 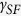 is the stacking fault energy corresponding to the (111) planes (e.g. Al*1* and Ti*1*, or Al *n* and Al *n* + *1*); *A*_*SF*_ is the area of stacking faults in area *A*. The stacking fault energies are assumed to be constant within the areas of stacking fault (bounded by the partial dislocations^30^). *E*_line_ is the total energy of the dislocation lines (blue lines) in the area A and is evaluated using 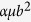. When evaluating the line energy of the misfit dislocations, the dislocations are treated uniformly as being edge type^31^, in which case *α* = 0.75. For dislocations in Al1-Ti1 plane (interface), the shear modulus 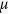 is taken as the average of c_44_ components of the stiffness matrix of Al and TiN transformed to the current coordinate system in Fig. 1(a), i.e. 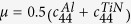; for dislocations in Al, the c_44_ component of Al is used. The spacing of the misfit dislocations only depends on the lattice mismatch between Al and TiN, and is invariant to the location of the misfit dislocation network with respect to the Al-TiN interface. Therefore, the interaction energies between the misfit dislocations is assumed to be constant with respect to the location of the MDN, and is ignored in the calculations of the excess energy. If the misfit dislocation network is away from the Al-TiN interface and inside Al, the Al (111) atomic monolayers between the misfit dislocations and the Al-TiN are assumed to be fully coherent with TiN lattice. Due to the large contrast of elastic constants between Al and TiN (Al being very compliant with respect to TiN) and the thickness of the Al monolayers being very small compared to TiN, the mismatch strain is assume to be partitioned into only Al. The strains in Al monolayers are calculated to be: *ε*_*11*_ = *ε*_*33*_ = 0.052, *ε*_*22*_ = −0.052. The strain energy density in coherent Al monolayers is evaluated using 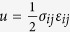. The Al*1* layer atoms have reacted with the N atoms and formed nitride structure with the same lattice constant with TiN, therefore the strain energy in the Al*1* layer is ignored.

## Additional Information

**How to cite this article**: Yadav, S. K. *et al.* Structural modifications due to interface chemistry at metal-nitride interfaces. *Sci. Rep.* **5**, 17380; doi: 10.1038/srep17380 (2015).

## Figures and Tables

**Table 1:** Excess energy of the MDN at different positions.

MDN plane	γ_SF_ (mJ/m2)	R (nm)	ISF percentage	Number of coherent Al monolayers	γ_exc._ (mJ/m^2^)
Al1-Ti1	1026	2.1	20.5	0	1936
Al1-Al2	332	1.9	2.1	0	583
Al2-Al3	16	39.7	48.7	1	457
Al3-Al4	111	5.7	40.8	2	587
Al4-Al5	80	7.9	43.4	3	618
